# Clinics of Interest at the N. D. A. Meeting

**Published:** 1901-11-15

**Authors:** S. H. Guilford


### CLINICS OF INTEREST AT THE N. D. A. MEETING.

BY S. H. GUILFORD, D.D.S.

At the annual meetings of the National Dental Association the interest of the great majority of those in attendance is not so much enlisted in the clinics and exhibits as in the papers and discussions. For this reason, probably, less effort is put forth to secure interesting demonstrations than would otherwise be the case.

Many of the clinics were interesting, but not many new ideas or devices were shown.

In the line of crown and bridge work, Millett's die-plate and swaging method attracted the attention of those who appreciate simplicity when linked with efficiency. It was an extreme simplification of the Hollingsworth system, and added interest was given to it by the fact that it was shown and demonstrated by Dr. Hollingsworth himself, who frankly conceded its improvement upon his own method.

Fusible metal, which plays such an important part in the Melotte, Hollingsworth and other systems, was not used at all. The labor of producing a gold shell cusp or facing was reduced to a minimum. The process consists in dropping a little ordinary sealing wax upon a short section of round brass rod, pressing it upon the desired form of the die-plate or natural tooth and chilling the wax in ice water.

The brass section with its impression is then inserted in one end of a brass cylinder; over it are placed the disk of gold and a roll of soft rubber, while into the other end of the cylinder a longer section of brass rod or plunger is introduced. The parts are now swaged in the ordinary way, and a good metal shell reproduction is the result. The entire operation greatly resembles the one employed for swaging inlays by the water-bag method. All is done in a few moments with of the simplest materials. Not only gold, but sheet brass, which is far more intractable, was swaged into perfect form without any tearing of the metal, which is such a common occurance where an intaglio die-plate or other metal form is used.

Probably the sealing wax yielded a little under the force of swaging, but not enough to be perceptible.

By this simple method one is not confined to the forms found on a die-plate, but the impression of the actual surface or face of a natural tooth can be taken and used in the same manner. The form in the sealing wax impression may also be altered to any desired extent by adding to or taking from it before it is placed in the swager.

If the simplifying process in crown and bridge work continues along these lines, it is difficult to tell to what narrow limits it may ultimately be brought.

Among the Association clinics very little was noticed in the way of new regulating appliances or methods, but one idea presented in a practical form was the Shryock countersunk nut.

In regulating appliances a nut placed beside some other part nearly as large as itself, as a tube, for instance, will cause the patient very little inconvenience; but when it happens to be located on the buccal side of some smooth surface it generally causes much discomfort by irritation of the soft tissues of the lip or cheek. We have had no good way of covering such a nut, although different expedients have been resorted to, and we could not round its sharp corners, else the wrench would not grasp it.

Dr. Shryock overcomes the difficulty by deeply counter-sinking the hole in the bar or band through which the screw passes and then constructing a tapering round nut to fit the counter-sink. To do this, the bar must be made sufficiently thick to allow the counter-sinking to be done without weakening it. When the nut is in position it does not project above the surface of the bar in which it is placed and as the screw begins to project by the tightening of the nut it is ground off, and thus all is kept smooth and comfortable.

The nut is turned by an instrument shaped like a screwdriver, with a deep notch ground into the center of the blade. This leaves two projections to fit into a slot in the head of the nut.

In those cases of irregularity where the malposed teeth are mostly located within the line of the arch, it is very common practice to force them from within outward by means of screws, springs, etc., but it is open to the objection that such devices occupy considerable space and thus interfere with speech if not with mastication. The opposite plan has been to spring a metal bow-wire around the outside of the arch (shaped to the ideal curve) and then by some means draw the malposed teeth outward until they come in contact with it.

For this purpose rubber bands have been used, but with their tendency to tear or slip and their operating less quickly than screws they have been employed under protest, as it were. Screws and nuts have seldom been used on account of the irritation to the mucous membrane which they cause.

The counter-sunk nut seems to overcome most of the objections heretofore existing to the use of the screw in certain positions in the mouth, and to that extent it is a valuable, even though a simple device.—*Stomatologist.*

